# A review of multi-factor authentication in the Internet of Healthcare Things

**DOI:** 10.1177/20552076231177144

**Published:** 2023-05-22

**Authors:** Tance Suleski, Mohiuddin Ahmed, Wencheng Yang, Eugene Wang

**Affiliations:** 1 School of Science, Cyber Security Cooperative Research Centre, 2498 Edith Cowan University, Joondalup, WA, Australia; 2 School of Science, 2498 Edith Cowan University, Joondalup, Australia; 3 School of Mathematics, Physics and Computing, 7932 University of Southern Queensland, Toowoomba, Australia; 4 Personalised Oncology Division, 5388 The Walter and Eliza Hall Institute of Medical Research, Parkville, Australia; 5 Faculty of Medicine, Nursing and Health Sciences, Monash University, Melbourne, Australia

**Keywords:** Multi-factor authentication, MFA, healthcare, IoT, IoHT

## Abstract

**Objective:**

This review paper aims to evaluate existing solutions in healthcare authentication and provides an insight into the technologies incorporated in Internet of Healthcare Things (IoHT) and multi-factor authentication (MFA) applications for next-generation authentication practices. Our review has two objectives: (a) Review MFA based on the challenges, impact and solutions discussed in the literature; and (b) define the security requirements of the IoHT as an approach to adapting MFA solutions in a healthcare context.

**Methods:**

To review the existing literature, we indexed articles from the IEEE Xplore, ACM Digital Library, ScienceDirect, and SpringerLink databases. The search was refined to combinations of ‘authentication’, ‘multi-factor authentication’, ‘Internet of Things authentication’, and ‘medical authentication’ to ensure that the retrieved journal articles and conference papers were relevant to healthcare and Internet of Things-oriented authentication research.

**Results:**

The concepts of MFA can be applied to healthcare where security can often be overlooked. The security requirements identified result in stronger methodologies of authentication such as hardware solutions in combination with biometric data to enhance MFA approaches. We identify the key vulnerabilities of weaker approaches to security such as password use against various cyber threats. Cyber threats and MFA solutions are categorised in this paper to facilitate readers’ understanding of them in healthcare domains.

**Conclusions:**

We contribute to an understanding of up-to-date MFA approaches and how they can be improved for use in the IoHT. This is achieved by discussing the challenges, benefits, and limitations of current methodologies and recommendations to improve access to eHealth resources through additional layers of security.

## Introduction

Authentication factors refer to user login credentials that a user supplies to an authentication process for it to decide whether to grant or deny access. When a user accesses their accounts online, it is of vital importance that their credentials are authenticated to ensure security. An authentication process involves the verification of the credentials a user is supplying to prove the user is who they say they are.^1^ Authentication technologies are changing with the emerging field of cyber-related Internet of Things (IoT).^2^ IoT-oriented authentication systems are being implemented rapidly across enterprises around the world, including the healthcare sector. Multi-factor authentication (MFA) provides extra layers of security, so in addition to a simple method of authenticating a user (e.g. password), additional verification, such as a one-time password (OTP), is sent to a user's email address or mobile device to generate a time-based code, meaning that at least two factors have been verified.^3^ Medical information is considered critical and sensitive. When this sensitive information is gathered by IoT devices, it is necessary that the channels of communication are protected from unauthorised entities during the transmission and storage of data.^4^ User authentication has progressed in the past decade from using single factors (e.g. passwords) to two or more factors to validate a user's identity so they can access services or data. In the healthcare domain, the systems being accessed, or the data being stored as records or transmitted by medical devices, are usually critical and sensitive. Therefore, ensuring the security of authentication systems is vitally important. This has become even more important in light of the COVID-19 pandemic, which has affected the world, so there is an urgent need for research into robust, lightweight security options in the medical arena.

Authentication involves multiple cryptographic approaches in developing MFA techniques, which can be integrated into healthcare-related IoT devices, enabling medical professionals and patients to protect critical medical information.^5^ However, this brings unfamiliar problems into the medical context of authentication security. Cyber attackers have relentlessly targeted the healthcare sector due to recent events in the world, especially COVID-19, which has put a strain on healthcare resources and caused an increase in cyber-attacks, impacting both patients and medical workers. Challenges for IoT devices in the medical context have become more prominent because the interconnectedness of equipment and devices allows attackers to move through systems using compromised accounts because of shortcomings in authentication security, such as weak passwords or repeated passwords.

### Related work

Several reviews have been conducted in the context of cybersecurity applications for Internet of Healthcare Things (IoHT) security. These reviews are related to our work as we identify the key security requirements for authentication systems for healthcare domain-based technologies and solutions. Altulaihan et al.^6^ reviewed the literature on cyber threats and risks to IoT security and categorised them according to layers of IoT architecture. MFA security classification is an important field, and its impact on authentication security can improve frameworks against potential cyber threats. According to Almaiah et al.,^7^ mobile users face various cyber threats to their privacy in interconnect networks. These risks are associated with the IoHT as they often rely on interconnected devices where information security can often be foreshadowed. Heterogenous networks are collecting various data from different users, devices, and network increasing their vulnerability over a larger threat surface.^7^ Hussain et al.^8^ explored researchers’ interest in the relationship between IoT networks and machine learning as an emerging field. As IoT networks develop, they grow larger and involve more social factors for both the users and the organisations implementing them. In authentication, privacy threats often arise as users’ locations are tracked by the devices or networks, they are accessing to authenticate themselves. Hussain et al.^8^ identified the threats to user location techniques, as adversaries can track important data or assets. Patient devices in the IoHT use various communication technologies, often centralised systems, creating an environment of risk for the user data should an adversary exploit a vulnerability. A decentralised approach to decentralising authentication model proposed by Almaiah et al.^9^ suggested deep learning techniques to distinguish authenticated users from adversaries in various IoT devices. The impact of security vulnerabilities in authentication models relying on traditional approaches and centralised databases for patient data privacy is critical for IoHT.

Additionally, remote access users are on the rise during pandemic restrictions around the world, increasing the demand for decentralised services. Siam et al.^10^ presented a healthcare technology for monitoring key security features in healthcare devices for sensitive patient data, which requires adequate security to safeguard user privacy. Kumar et al.^11^ proposed a novelty approach for a framework of IoHT smart healthcare systems through anonymity using the blockchain technology. Blockchain offers security benefits through privacy preservation by managing a network of IoT authentication infrastructure.^11^ Blockchain in the IoHT context can enhance the security of patient health record storage, and frameworks have been proposed as an approach to implementing trust chains for data access.^12^ Trust chain technology is a cost-effective solution for scalability, which is important in the IoHT, because the large volume of interconnected networks often relies on centralised systems. Centralised systems for healthcare providers are vulnerable when an adversary breaches security, so there is a demand for privacy assurance whenever sensitive data is involved.^13^ Almaiah et al.^14^ presented deep learning-based methods for privacy preservation in industrial IoHT frameworks. This scheme uses verification and validation of entities accessing data on IoT devices and then applies deep learning to intrusion detection.

Another relevant field is mobile networks, which is important for facilitating a vast proportion of data exchange through interconnected networks, where data exists in many forms such as texts, images, or audio.^15^ Almaiah et al. proposed a scheme for encryption of cryptographic key exchanges in networks, which is beneficial for improving the security features of passwordless authentication solutions. Smartphones present a major security challenge for authentication, as cyber threats continue to emerge, and users access a lot of sensitive information on their mobile devices.^16^ Bubukayr and Almaiah^16^ reviewed the cyber threats and their respective countermeasures, like those used in IoT authentication security. Wireless sensor networks (WSNs) are employed in several industries, including transportation, healthcare, smart buildings, and smart cities. Almaiah^17^ proposed a blockchain approach for attack detection as an appropriate countermeasure. The proposed idea uses peer-to-peer techniques to establish a distributed network, which suites IoHT environments, providing better privacy management amongst devices.^17^

Research shows that IoT networks suffer from resource-constraints, as networks communicate a variety of healthcare data and can easily become oversaturated with new devices.^18^ Khan et al.^18^ proposed to use the MAC address and location in neighbouring nodes of WSNs to secure data through verification before sending it to the next node. This algorithm can secure large networks in IoHT environments. Moreover, aggregated data management is useful for smart city mapping. Smart cities seek to improve infrastructure for data storage, processing, and transmission.^19^ The IoT is utilised to accommodate services and applications for a wide range of data management, sharing common ground with authentication strategies against cyber threats.^19^ A well-known approach to dealing with resource constraints in IoT environments is through cloud-based services. Cloud-based authentication can reduce overhead and improve cost efficiency by distributing systems across large, interconnected networks made up of various devices.^20^ Cloud-based security features enable enhanced countermeasures to be deployed across complex environments, which is desirable in the IoHT with remote access demands increasing. Secure systems for cloud computing in IoHT domains are vital for protecting sensitive patient data such as heart rate, temperature, and blood oxygen levels, all of which can result in life-or-death situations should an adversary compromise them.^21^

### Research motivation and contributions

While the existing related work above provides insights into cybersecurity applications for IoHT security, remote user access control, mobile network security, etc., to the best of our knowledge, existing solutions of MFA in the IoHT inadequately identify the key security requirements of next-generation authentication applications. There is a very little survey available for the passwordless approach to MFA in IoHT domains. Moreover, the COVID-19 pandemic shifted the paradigm of working environments in healthcare, and it is important to review how existing MFA solutions affect IoHT. To fill this gap, this review paper evaluates existing solutions in healthcare authentication and provides an insight into the technologies incorporated in IoHT and MFA applications for next-generation authentication practices.

The main contributions of this paper are emphasised as follows:

- *A comprehensive review*: Authentication technologies in the existing studies and their relationships are analysed comprehensively in the scope of this paper. The existing literature is examined to identify the security and research gaps in the current technology in light of the increased demand for IoHT devices and the need for robust and lightweight security processes. Regarding authentication, we investigate the cyber threats to MFA techniques and identify the security requirements for healthcare applications. Furthermore, this review identifies key aspects of the COVID-19 pandemic and its effects on authentication practices in healthcare domains. As authentication shifts from primarily organisational resources to work-from-home and bring-your-own-device, the security challenges are discussed in this review paper.
- *Identifies the research gaps in current MFA solutions*: This paper covers the impact and challenges of applying current MFA techniques in healthcare. Many papers in the literature tend to discuss the security challenges observed in settings. However, this review paper identifies the research gaps through a systematic literature review based on the keywords of ‘medical IoT’, ‘medical biometrics’, and ‘medical authentication’ to ensure relevance and scope. We discuss the cyber threats to IoHT authentication and forward-thinking principles to approach the challenges in healthcare authentication studies.
- *Insights to future directions of authentication in a medical context*: Results of existing research and currently implemented technologies are discussed and analysed. Based on the results, the authors point out several future MFA research and development directions on IoHT devices based on the identified security requirements.

### Research criteria

Papers are eliminated from the collection of the reviewed articles according to specified keywords if they are not relevant to the research parameters defined. At the time of authoring this paper, we reviewed the literature published over the last decade (2011–2022) as our scope for the transition of IoHT. This paper aims to bridge the gap in security requirements and strategies of authentication systems in the IoHT by discussing the healthcare taxonomy for IoT devices. The literature refers to the following databases for a wide range of related work: IEEE Xplore, ACM, JMIR, ScienceDirect, SpringerLink, MDPI, and various conference papers. The purpose of the broad range of databases in this paper is to ensure there is a holistic overview of state-of-the-art MFA solutions to identify the key security requirements and features for next-generation authentication schemes.

**Figure 1.**
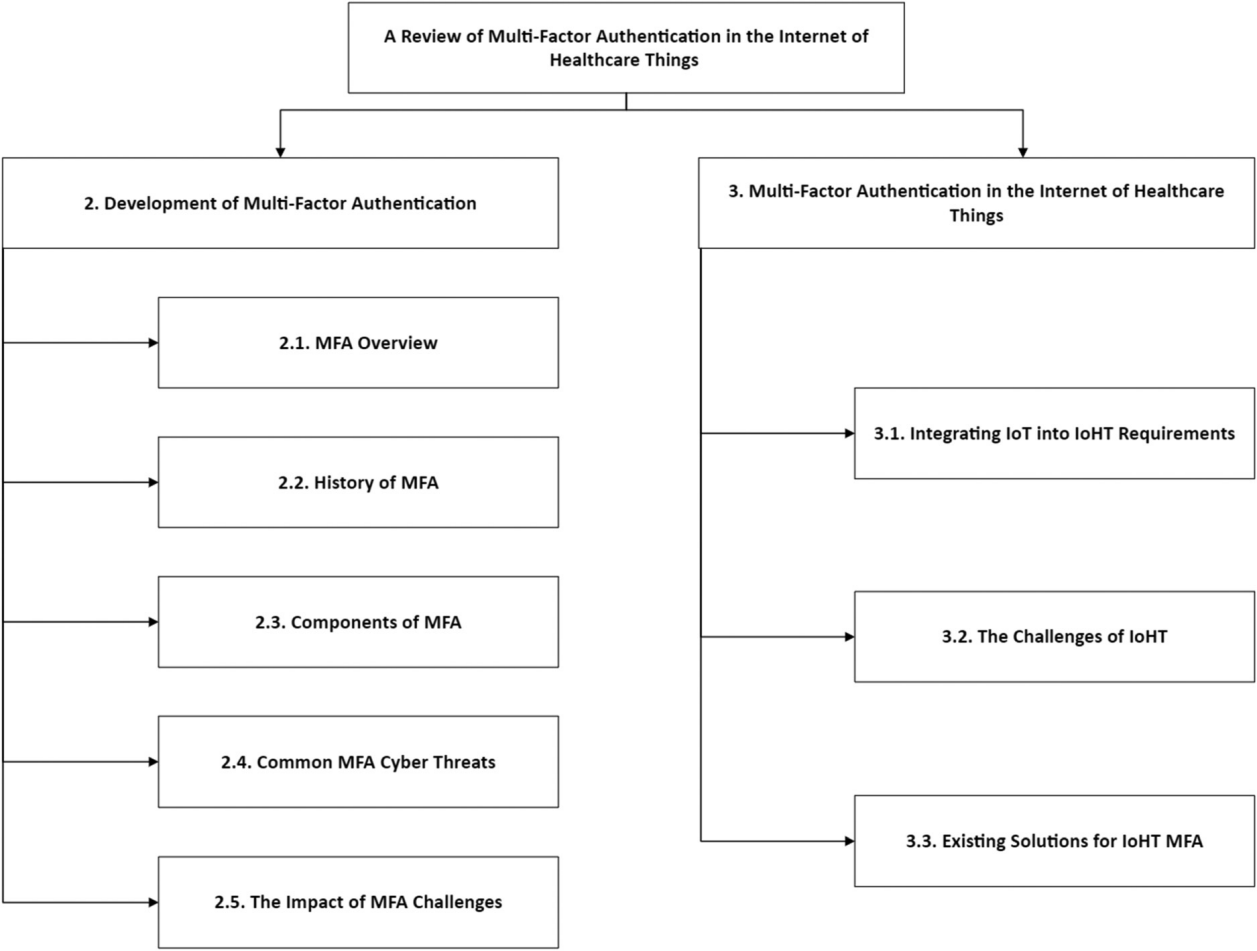
Taxonomy of the two-fold objectives in this paper based on MFA and IoHT literature.

MFA: multi-factor authentication; IoHT: Internet of Healthcare Things.

The literature review considers the stance of advancing modern authentication systems to adopt secure, robust, and lightweight improvements over traditional methods, so that weak password practices can be strengthened when handling sensitive patient data. The review is arranged as follows: the Introduction section presents MFA development and the current challenges and solutions that exist, while the ‘Development of MFA’ section explores healthcare domains and the development of IoHT because of MFA, as in Figure 1. The remainder of this paper is organised as follows. The ‘MFA in the IoHT’ section discusses the challenges of IoHT and existing solutions for MFA in IoHT. The ‘Conclusion’ section concludes this paper and recommends future research directions on MFA in healthcare.

## Development of MFA

### MFA overview

MFA involves various authentication principles applied to the login process of a system through multiple devices by gathering enough evidence to verify a user is who they claim to be. The source of these access points can come from human interaction with a system, whether it be trust-based systems, knowledge-based systems, or any method of credential-sourced technologies to allow a user to prove they are the legitimate user of a system. Often passwords are used in combination with two-factor authentication (2FA) or MFA, namely two or more factors of authentication are used to enhance the security of the credentials being used.^1^ Some examples of 2FA are passwords, PIN codes, biometric traits, and memory cards, each belonging to their own respective categories based on the ‘type’ of factor.^1^ Therefore, factors of authentication methodologies are categorised based on knowledge, possession, and inherence. These categories are defined in Table 1 with their methodologies and their application in present systems. MFA requires two or more subsets from these categories, while single-factor authentication (SFA) was traditionally developed with the criterion of a knowledge-based factor.^22^

**Table 1.** Factors of authentication components^1,23^.

Factors	Definition	Methodologies
Knowledge	Authentication factors that a user can remember based on alphanumerical codes kept private to the entity or group of entities interacting with a system, such that the user has knowledge of the required factor.	Passwords Security question/answer combinations PIN codes
Possession	Devices or physical objects that often contain a combination of hardcoded credentials to authenticate the user, for example, a security key or type of card that can be scanned to automatically apply public key cryptographic exchanges with authenticators, which have the pairing private key.	Physical keys USB Mobile devices OTPs Smartcards
Inherence	Biometric traits or elements that consist of human behavioural credentials such as voice patterns or even a human signature, which are unique and often hard to impersonate. Biometrics are unique attribute-based factors that belong to a user and are much more difficult to replicate than their object- or knowledge-based counterparts.	Fingerprint recognition Face recognition Voice recognition Iris recognition Signature recognition

OTP: one-time password; USB: universal serial bus.

*Note*: A combination of factors is used in security systems to authenticate a user and ensure the user is legitimate and not an adversary or unauthorised user masquerading as an authorised user. Often, the application of each factor and its sub-factors is based on the system requirements and data being handled.

### History of MFA

MFA improves the functionality of SFA and 2FA by providing additional layers of security that an adversary must obtain to masquerade as a legitimate user. These additional factors ensure that the MFA system meets an organisation's security requirements and obligations to ensure privacy. However, studies show that oversaturating security can lead to other issues such as complicated security procedures that result in poor user security posture and a lack of awareness of good practices.^24^ Usernames and password combinations are still used as a basic approach^24^ to handling user authentication in most industries around the world without complex systems. Complicated authentication systems often lead to an increase in bad practices because users may be tempted to leave evidence or clues as to what their passwords are on their desks or use public information such as their date of birth in their passwords.^25^ On the other hand, as most information is collected, transmitted, or stored digitally, easy-to-guess or easy-to-crack passwords become less reliable and susceptible to cyber-attacks such as brute-force attacks,^26^ which are the most common due to their simplicity and reasonable requirements for computational power. A brute-force attack can be launched with rudimentary knowledge about computer and information systems, so it is not regarded as a sophisticated attack given that it attempts to guess all possible password combinations of a user through easy-to-acquire software tools.^27^ Cyber-attacks are a common example of password cracking, and the extent of the attack tools an adversary can use is discussed in detail in the ‘MFA in the IoHT’ section with new emerging platforms as cybercrime increases globally.

2FA is an accepted approach to securing user data^28^ and providing additional defence against brute-force attacks, dictionary attacks, snooping, or man-in-the-middle (MITM) tactics by adding a factor of possession (e.g. a smartphone), which acts as an extra step in the authentication process. Petsas et al.^29^ conducted a study on the practicality of 2FA on Google accounts to analyse the performance of increased security measures. The study found that only 6.4% of the 101,047 Google accounts adopted 2FA. In practice, the implementation of 2FA requires the use of a smartphone, an email address, or a key generator as an additional security layer together with the users’ knowledge-based factors like their passwords or PIN codes.^30^ However, using human entities as additional factors is known to be problematic,^28^ since automated attacks can take advantage of fraudulent authentication mechanisms to authorise illegitimate users once username/password combinations are compromised.

Rivest, Shamir, and Adleman devised an algorithm known as RSA^31^ for public key encryption and private key decryption for authentication purposes. However, the weakness of RSA is that it is a time-consuming process, which decreases cost efficiency. Additionally, Alamsyah et al.^31^ proposed a methodology to assist with asymmetric key algorithms. This approach combines a 2FA approach using OTP to strengthen security efforts against common cyber-attacks.

**Figure 2.**
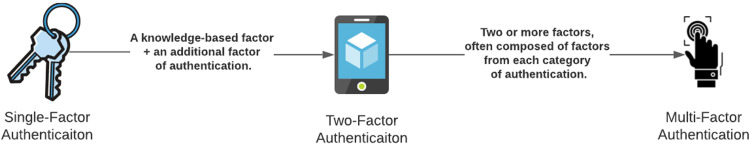
Interpretations of the development of MFA from SFA to MFA. *Note*. Based on the findings,^22^ this figure shows how SFA is developed into 2FA using conventional methodologies from knowledge-based factors combined with an additional factor, such as mobile OTPs.^24^ While MFA covers principles of 2FA, it is commonly recognised to be a more advanced standard in that it provides additional layers of security by making use of mobile devices built with lightweight capabilities suitable for wireless networks or cloud systems.^32^

MFA: multi-factor authentication; SFA: single-factor authentication; 2FA: two-factor authentication; OTP: one-time password.

MFA involves various complex techniques, aiming to improve overall security measures and meet stringent requirements for handling critical and sensitive information/data, such as data in medical contexts.^30^ MFA offers an easy-to-access solution to most organisations, given that the additional factors required for authentication are often devices familiar to users, such as their mobile phones or hardware tokens, and hence require no advanced training or understanding on the part of the user.^30^ The development of MFA is illustrated in Figure 2, which depicts the advancements from SFA to 2FA and then to MFA.

### Components of MFA

In this section, we discuss the current literature, research, and development of MFA in healthcare and how the factors (or components) are applied to MFA techniques. The addition of cryptographic algorithms improves SFA using different techniques, such as signatures and public–private key pairing, all of which can be combined to strengthen passwords in the authentication process. Modern concepts of MFA utilise components made up of each category of factors to suit the requirements of an organisation so that the risk of information leakage or data loss is mitigated.^30^ In the following section, we categorise these components into their respective authentication factor categories to map the architecture of MFA with a focus on healthcare applications. The information in this section lists the different components and we discuss the security requirements of the respective methodologies as detailed in Table 2.

**Table 2.** Components/mechanisms of existing MFA.

Categories	Components	Methodologies	Source
Knowledge	What you know	Password management Digital signatures	^25,33–35^
Possession	What you own	OTPs Physical keys/smartcards RFID NFC Implantable/wearable devices	^30,34,36–40^
Inherence	What you are	Biometrics Behavioural biometrics Biometric data (ECG, fingerprint veins, etc.) Artificial intelligence Monitoring devices	^23,26,27,41–46^

MFA: multi-factor authentication; OTP: one-time password; RFID: radio-frequency identification; NFC: near-field communication; ECG: electrocardiogram.

**Figure 3.**
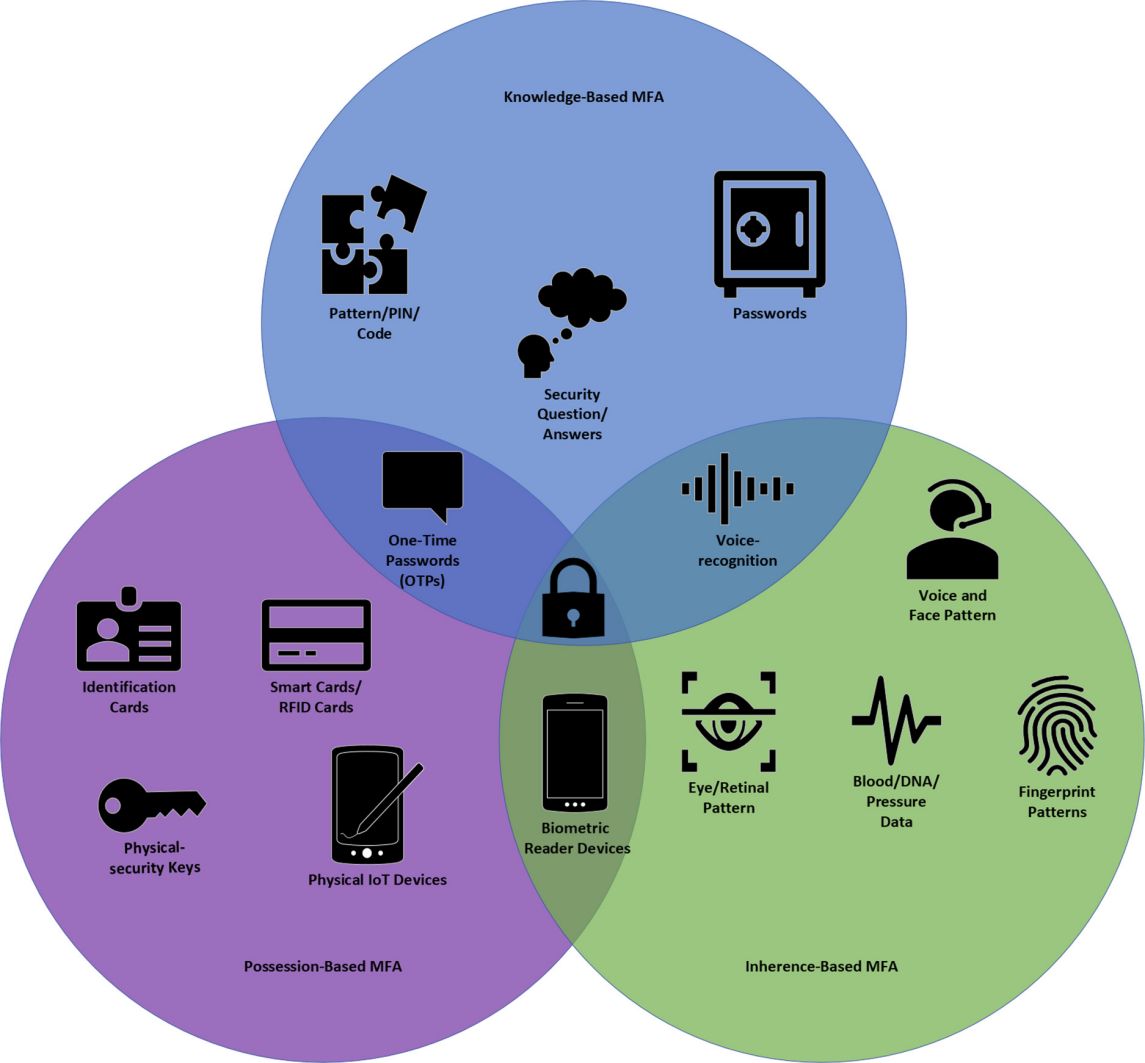
Venn diagram of current authentication factors/components in multi-factor authentication (MFA).

Existing architectures for the IoT authentication domain present guidelines to support security measures to address the most common forms of attacks that a component of MFA may suffer, thus adapting existing technologies to a certain environment.^47^ Novel solutions in relation to authentication factors have been recommended by research communities to ensure better security for credential handling and authentication systems.^1^ Solutions or best-practice methodologies include complex passwords to minimise brute-force attacks. Figure 3 illustrates the three-factor categories with examples of their applications in existing real-world solutions where the components of each can be applied. We note that some factors are hybrids and have their own subsections within two or more categories as technology advances and security requirements can be managed using an integrated approach.

#### What you know

What you know corresponds to the knowledge factor, this category contains authentication factors that are often created by a user. The factor can be patterns, phrases, alphanumerical or special characters created in combinations or sequences to create something that can be remembered by the user.

*Passwords*: Passwords are the most common choice of authentication. A lot of research in the domain of MFA revolves around adaptations of passwords to mitigate conventional attacks like brute-force attacks or password cracking.^25^ Therefore, password handling improves when it is combined with other security measures. Adding extra steps^35^ that not only a user but also an attacker needs to take in the authentication process can better verify that the user is who they claim to be and deter attackers from compromising security. The current literature on MFA in healthcare shows that password auditing on weaker systems or smaller organisations can reduce threats and defend against security breaches. Tools for password auditing^35^ raise awareness and assist in developing better protocols and policies towards maintaining security; however, cyber-attacks can still take place even when using MFA, as discussed in the ‘Common MFA cyber threats’ section.

*Digital signatures*: Digital signatures enable static authentication on IoT devices tailored to many users compared to passwords. Conventionally, a password must be set and repeated by a user on each login, however, a digital signature is a time-efficient and cost-effective alternative^33^ in secure systems. A digital signature can be configured into IoT devices and governed with automated scripting and policies for maintenance, making them relevant and available. Should a user no longer be authorised to use a specific system, they can easily be removed. Digital signatures can be encrypted to mitigate the risk of being divulged to cybercriminals.^33^ Secure environments can further improve the security posture of a system or network. For example, digital signatures set for unique users can be monitored and prepared in a controlled environment, allowing patients to access healthcare services in a timely manner^34^ without using password-based credentials.

#### What you have

What you have corresponds to the possession factor. This category often involves hardware because physical objects are what a user must have or own to prove their identity. However, considering healthcare environments, not all hardware solutions are restricted to possession factors. OTPs are delivered logically through a mobile device or secondary point of contact, for example, through an email that a person has, with the knowledge only they should have, such as the username/password combination for that email.^30^ OTPs ensure a new code is generated on each login and help to deter or stop adversaries from accessing accounts remotely by posing as an authorised user digitally and illegitimately. OTPs can also be configured into physical keys or tokens as possession-factor integration for MFA; however, this does not prevent duplication attacks on the physical token itself^30^ without a sensor to monitor or audit the functionality of a smart card.

*Short-range communications*: Radio-frequency identification (RFID) authentication technology is usually applied to possession-based factors, such as smart cards or security tags, and implemented into IoT devices for identity control.^38^ RFID and near-field communication (NFC) are localised technologies,^36^ which provide access control to physical locations and allow for prompt user identification via close-ranged communication channels to reduce the persistent threat of remote hackers. RFID cards can be used to authenticate one tag per session, known as per-tag identification.^38^ Unidirectional authentication is a technique for protecting data privacy in RFID systems. In conjunction with OTPs, tags can be augmented with asymmetric cryptography to enable remote user identification^34^ by storing the public key within the device and interacting with private keys within RF readers.

*Implantable medical devices (IMD) and wearable devices*: IMD and wearable devices are IoHT devices and are becoming commonplace in healthcare settings. They are often used in monitoring systems and sensor analysis so that contactless approaches and persistent services to patients can be provided, ensuring a patient's medical data is kept private, available, and accessible remotely.^40^ These devices allow for large data-sharing operations between multiple hardware components, such as smartphones, tablets, and display units. Compatible with MFA, the devices can be configured to allow the use of biometric factors in combination with the device's physical mechanisms.^37^ In authentication, this can be used to supply an additional factor where biometrics or push notifications can be sent to a user's wearable device to act as a security layer to verify the person is who they claim to be. IMD can be used in a similar scenario where authentication can be automated based on the data that is being purposed from the device to match the patient's biometric data as authentication. Examples of IMD and wearables are implanted pacemakers or insulin pumps, which can be connected either internally or externally on a patient to store or transmit health data to monitoring devices.^37^ These applications help to improve healthcare by reducing the cost of monitoring and examining patients during their treatment or rehabilitation using autonomous systems in interchangeable devices. IoHT devices of this nature depend on technologies such as wireless access or wireless body networks and are therefore vulnerable to many known attacks and suffer weaknesses known in other IoT device configurations.^39^ We discuss this further in the ‘Common MFA cyber threats’ section.

#### What you are

Components regarding what you are come from the inherence category as they are factors of unique traits and characteristics with which you were born, so they are much harder to replicate or clone, unlike hardware possession factors.

Cryptographic improvements to biometrics^27^ have changed how organisations, including healthcare, handle their data storage, transmission, and collection techniques. Based on the unique identification process of inherence factors, biometric cryptography can be used to overcome the limitations and weaknesses of SFA, such as issues of weak passwords or PIN codes.^27^ Using behavioural biometrics for smartphone users, authentication can be performed through a user's signature, keystrokes, and voice or touchscreen interactions. Profiling such behaviour^23^ forms biometric solutions for IoHT devices. Mobile phone biometric authentication is utilised by many research and development communities as an approach for user authentication, based on fingerprint or face recognition, which can be found in most modern mobile designs, allowing for easy-to-use, lightweight solutions.^42,44^ Another solution for a cancellable finger vein-based bio-cryptographic system^46^ not only offers user authentication but also allows the encryption of sensitive medical data through a biometric encryption technique called fuzzy commitment. Another interesting approach is the utilisation of electrocardiogram (ECG) technology^45^ for user authentication, which is based on the patterns of users’ heartbeats,^45^ so ECG signals can improve MFA by moving from conventional SFA or 2FA of simply a password and email/SMS combination. Instead, existing technologies common in healthcare practices (e.g. ECG) are being explored to improve authentication security and ensure security requirements are being met based on the changing climate of IoHT.

*Monitoring systems*: Healthcare services should provide an ecosystem to look after patients’ needs by utilising smart systems. Monitors can be embedded in or worn by patients, allowing user authentication to be performed between patients and their attending medical staff who oversee their medical data.^43^ Through advancements in deep learning and artificial intelligence (AI) for smart monitoring systems,^43^ it is possible for authentication to be governed by AI, which facilitates complex computational resources to protect the security and privacy of patients’ information while ensuring such information is available to patients and medical staff anywhere and anytime. Users are authenticated through their unique biometrics at the discretion of AI to determine discrepancies and measure the validity of the authenticating components used. This means that AI can be trained through deep learning^43^ to filter fraudulent attempts using real-world data. Attribute-based biometrics is a promising direction for establishing additional layers of security in authentication processes through AI.^26^ MFA in medical contexts follows various disciplines and applications of data collection in IoT devices. A solution proposed for attribute-based frameworks^41^ protects user privacy when users interact with IoT systems, preventing their identity from being misused or traceable and decreasing the attack vectors present.

### Common MFA cyber threats

Due to a lack of understanding of an organisation's security measures utilising IoT technologies, user authentication suffers from a variety of cyber-attacks. Cyber-attacks are easier and cheaper to assemble than physical attacks, as they can be performed remotely and therefore often go unnoticed^48^ until data is lost, destroyed, denied, disrupted, exfiltrated, or manipulated. The following subsections are by no means an exhaustive list of cyber-attacks, but an indication of some common attacks. Challenges to MFA are likely to impact research and industry standards. It is known that some complications impact not only research communities, developers, and vendors but also security rules of organisations and how these technologies are implemented in practice. Forgetting a ‘strong’ password often causes employees to choose easier or repeated passwords. Improving the strength of passwords tends to be motivated by increasing the time and resources that an attacker must dedicate to crack a password. Although MFA techniques are an improvement on knowledge-based password generation, MFA procedures can lead to human error and poor application^49^ of the policies in a workplace.

#### Brute-force and dictionary attacks

Brute-force and dictionary attacks have been developed over the past decade as simple methods for cracking passwords by attempting every possible combination until access is granted and authentication is successful. As the concepts behind these attacks became well-known and better understood by security communities, so did the approach that attackers took to evolve their efforts using botnets^50^ to crack passwords with prevalent force against simple systems.

#### Communication-channel attacks

**Figure 4.**
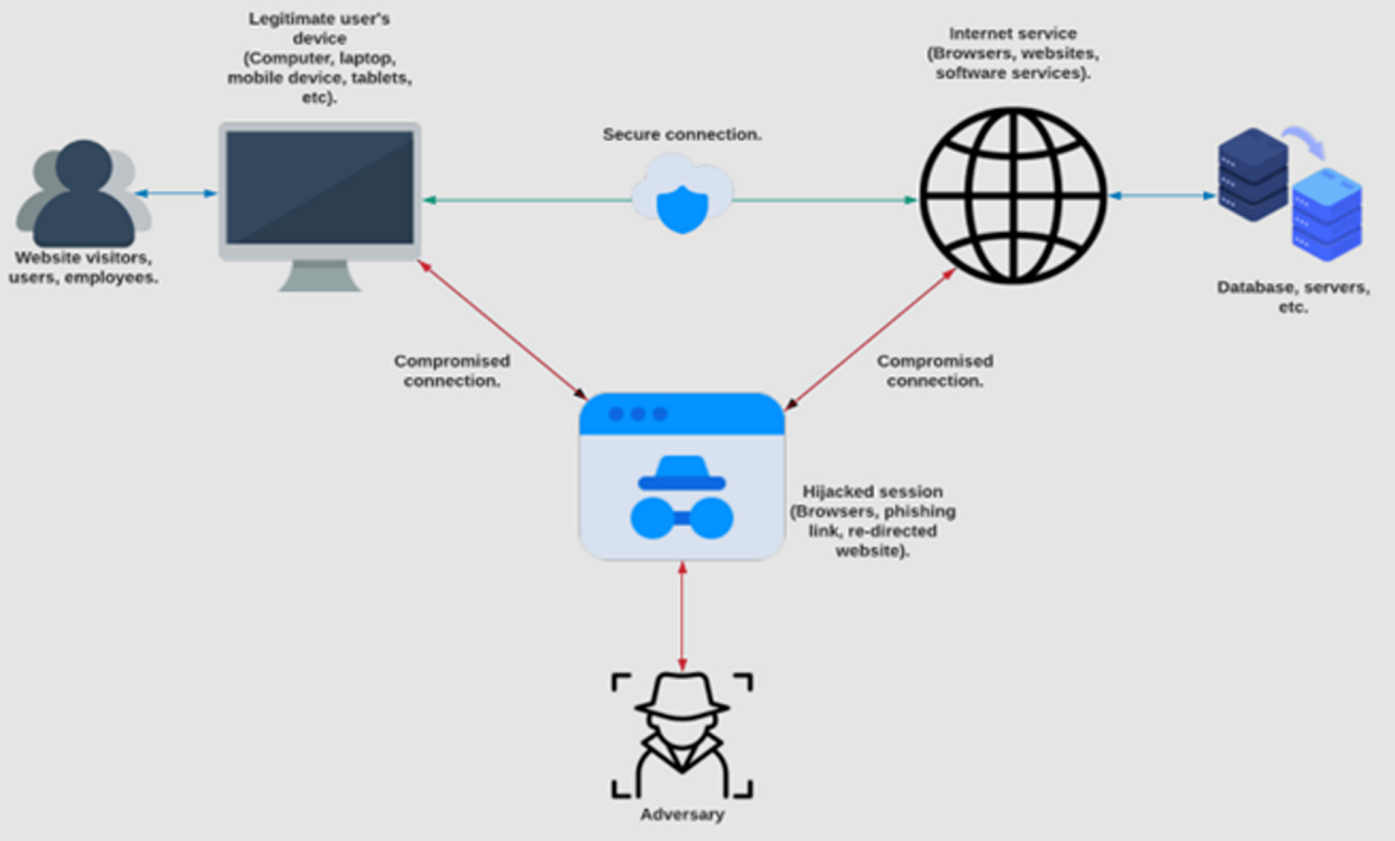
Illustrates the secure channel of a legitimate user who has authenticated themselves on a device and is interacting with a service such as data through a browser or server being manipulated by an adversary through the man-in-the-middle (MITM) attack type. Note. This adaptation of a MITM attack is based on the key objectives of the author.^51^ This figure includes details associated with the interception of secure connection data, allowing an attacker to inject themselves within a secure connection and further manipulate a session.

*MITM attacks*: A MITM attack is usually set up remotely by an adversary to intercept the line of communication between users or systems. MITM attacks are often involved in cyber-attacks on authentication structures.^51^ For example, in the healthcare setting, a MITM attack scenario is classified as a high complexity attack,^34^ as the adversary would need to have physical access to the communication channel or network. The adversary intercepts the communication channel between two legitimate entities, such as a patient on their device and a healthcare service like a portal for accessing medical records,^52^ as shown in Figure 4.

*SQL injection attacks*: A code injection type of attack is often used for infiltrating websites. These attacks can take place by escalating the privileges of the user for root or admin access to the system to bypass security measures, or by establishing an illegitimately authenticated user in the network.^53^ Impersonation attacks work together with MITM attacks, as shown in Figure 4, as the adversary attempts to replicate legitimate sites, such as webpages, portals, etc. for health services. These attacks also involve malicious payloads that can hijack a session and allow the adversary to stay logged in as the legitimate user when they supply their credentials on the fraudulent site.^34^

#### Social engineering attacks

*Phishing*: Social engineering against authentication is an easily accessible attack with low skill requirements and can be executed by a novice adversary. It is a technique to manipulate human behaviour and bypass most information system security efforts.^54^ Social engineering entails various approaches to steal the credentials of a legitimate user, with phishing attacks being the most notable. Attackers employ various social techniques to pose as a legitimate entity to create a communication channel and deliver malicious attachments, often obscured as urgent/important files, images, or software with malicious payloads.^54^

*Spear phishing*: This attack exploits a multitude of vulnerabilities in MFA, because the adversary can leverage their approach to target specific staff (e.g. medical doctors) based on their position or title. By compromising a staff member of a higher status, adversaries can masquerade throughout the system and interact with many users at ease, as they have access to more parts of the overall network.^55^

### The impact of MFA challenges

Challenges to MFA are likely to impact research and industry standards. It is known that some complications impact not only research communities, developers, and vendors but also the security rules of organisations and how these technologies are implemented in practice. An employee who forgets a password using best-practice approaches to password strength can often resort to bad practices, such as using easier or repeated passwords after they are reset, increasing the risk to the organisation's security. Improving the strength of passwords tends to be motivated by increasing the time and resources that an attacker would have to dedicate to crack a password. Although MFA techniques are an improvement to knowledge-based password generation, MFA procedures can lead to human errors and poor application^49^ of the policies in a workplace. The current climate of the COVID-19 pandemic is only one of many challenges faced by the healthcare sector regarding IoT technologies. In this paper, we discuss the key components of the cyber threats to MFA in relation to the design and approach taken to address security requirements to gain a better understanding of managing MFA in healthcare. IoHT inherits many challenges from IoT such as the security requirements of smaller mechanisms with constraints in relation to resources and the development of authentication devices. We discuss this briefly as we focus on the main objective of establishing the key security requirements, which impact the development of authentication methodologies from the past decade of literature.

Therefore, the impact of cyber threats that persist in these industries, especially in healthcare where many IoT devices are connected to one another, is notably higher.^56^ Al-Qaseemi et al.^56^ wrote that many research communities cannot agree on a concept that works best at each individual layer of IoT. The standards still lack the security requirements of modern climates as MFA continues to grow rapidly due to the high demand from the healthcare sector affected by COVID-19.^56^ Cybercriminals put further stress on healthcare systems, which are already struggling to provide treatment for patients using ransomware,^57^ which denies a user or organisation access to their files unless they pay a ransom. In healthcare organisations, this trend in cybercrime is causing public and private firms to become targets for further ransomware attacks that increase in complexity.

## MFA in the IoHT

The global coronavirus pandemic has affected many people's lives and has tested the limits of the healthcare sector. To explore the potential development of authentication techniques for the healthcare sector, it is important to understand the challenges faced by hospitals, clinics, and healthcare-related organisations. What are feasible and acceptable approaches to securing sensitive information, such as patients’ health records and data? How can we secure the resources needed by healthcare industries in their supply chains? Primarily, we must ensure authentication methods are robust, easy to use, and acceptable to their intended users. There is a strong demand for policy arrangement alongside training and awareness for healthcare workers when handling sensitive information, as cybercriminals can deploy an array of attacks^58^ to compromise or breach security where healthcare information is divulged, and healthcare services are destroyed or disrupted. Healthcare is at constant risk of cyber-attack because adversaries target medical records of patients or even control the dispensing of medicines and the utilisation of medical equipment^59^ in denial-of-service attacks in attempts to bypass security measures. Cyber criminals often launch attacks against healthcare industries due to financial motivations; for example, medical records contain identity and other sensitive information of patients that are of high interest to cyber criminals.

MFA is crucial for the future direction of the healthcare sector. It is a development trend to replace traditional authentication methods by going ‘password-less’ so that the threats of exploiting social knowledge-based factors^60^ can be mitigated. Social networking is growing rapidly as the availability and ease of access to platforms increases. The sense of safety that users have is increasing too, when sharing something on public networks. However, the issue is that the information shared about a person when made public in social contexts is likely to contain hints or answers to security questions adopted by MFA techniques to support passwords.^60^ The COVID-19 pandemic has forced us to work from home or remotely access an organisation's resources, but the lack of strict security policies or surveillance from an organisation makes it a target for cyber criminals who can take advantage of these weaknesses.^61^ Therefore, to understand the severity of cyber-attacks, it is important that users conform to the best-practice authentication security solutions provided by their organisations. Also, organisations should increase the training and awareness of the users who interact with healthcare resources and information, especially those who are working remotely from home.

During the pandemic, cyber criminals focused their attention on healthcare to disrupt services through ransomware attacks. They spread mass phishing emails to healthcare workers by exploiting covid-related strategies to deceive users into opening links, thus leaving their accounts vulnerable.^62^ As hospitals are a critical infrastructure and play a significant role in controlling the pandemic, it is vital to ensure that future cyber security policies provide funding to compensate hospitals for the costs associated with increasing the security of hospitals’ authentication systems. While the healthcare sector has long been a target of cyber criminals, the surge of attacks targeting patients’ personal data and medical records is a concern for the cybersecurity posture in healthcare-related industries. A well-known cybersecurity company, Bitdefender, that provides security solutions reported a 60% increase^63^ in phishing attacks on hospitals during the pandemic, especially in March 2020 when the pandemic had begun to spread globally.

### Integrating IoT into IoHT requirements

**Figure 5.**
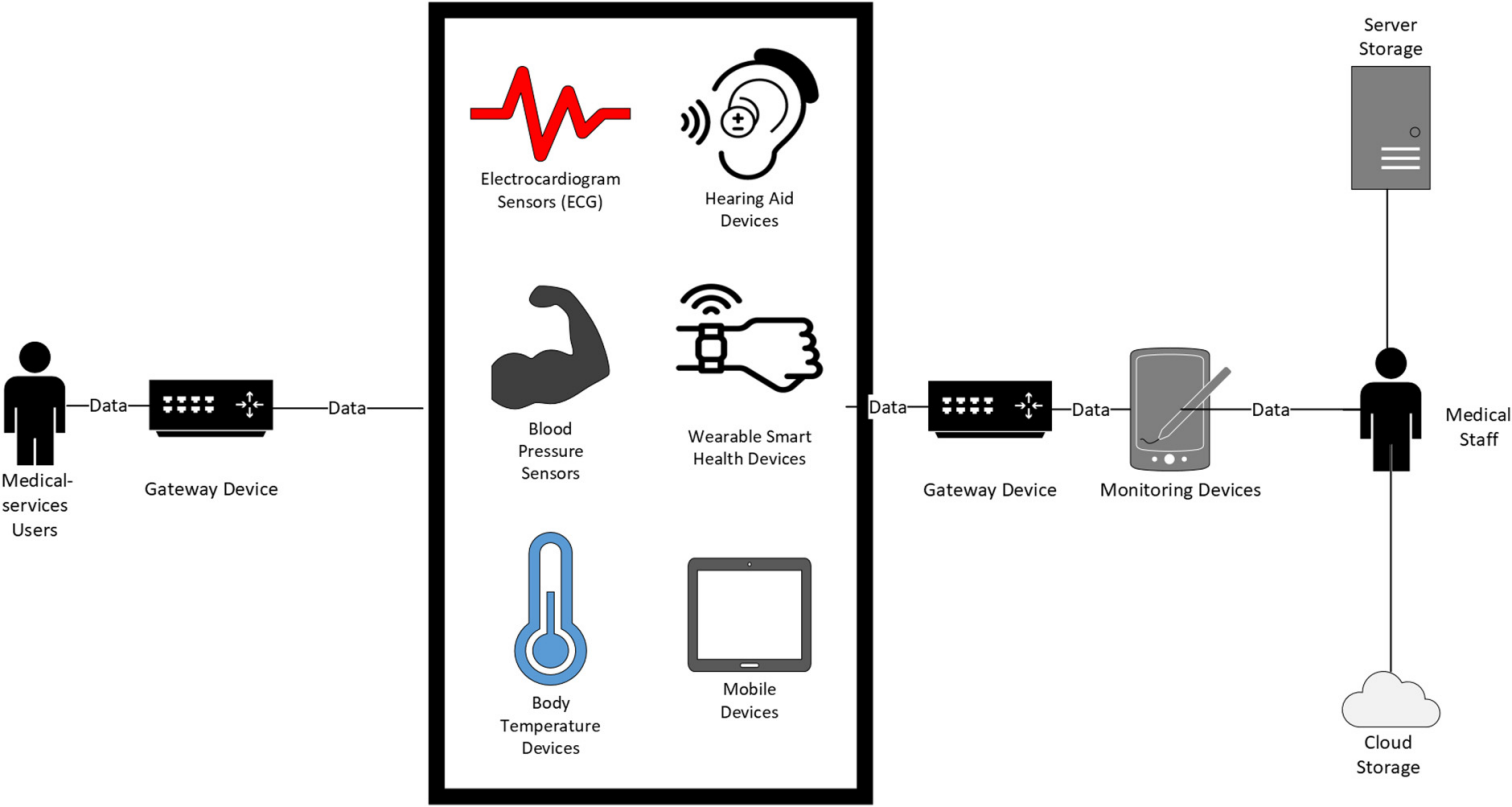
Internet of Healthcare Things (IoHT) data management between devices, users, and systems in the healthcare sector. *Note*. This figure is adapted from Shrimali^67^ and depicts healthcare sensors and relevant IoHT data in motion between different IoHT applications, showing that data moves between patients and medical staff in a typical healthcare environment. The data is then either moved, stored, or used between the different IoHT devices and processed through servers or cloud technologies.

As discussed in this paper, the IoT is a growing component of digital information and the integration of systems and networks to expand the usability of technology with digital data. As healthcare relies heavily on these technologies, we have seen a rise in the IoHT as its own domain and it faces unique challenges, requirements, and approaches to a novel solution, which can meet security and authentication demands. The IoHT is concerned with technologies that actively and passively interact with patients’ confidential data that is either stored, transmitted, or processed. A critical requirement for the IoHT is the configuration and utilisation of IoHT devices^64^ in current medical practices for better security and user authentication. It is desirable to strengthen security at the device-level,^65^ not only improving the design of IoHT devices but also raising the security awareness of device users. Vulnerabilities exist in the portability of IoHT devices due to their purpose as wearable or implantable sensors, working in real time to transmit data^52^ from patients to monitoring systems. The risk of IoHT devices being hacked by remote adversaries could be serious in a time-critical situation, where a patient's life is on the line. Therefore, IoHT specialists are in high demand around the world when markets push the benefits of IoHT technologies to the healthcare sector. It is inevitable that the scope of attacks on IoHT data will increase as more IoT devices are becoming IoHT-oriented,^66^ as shown in Figure 5.

Research on IoT networks has made lightweight advancements in healthcare settings of IoT technologies, such as two-way two-stage authentication protocols,^68^ integrating nodes to store sensitive information (e.g. patients’ medical data) in a way that it cannot be tampered with. Establishing efficient and secure MFA systems requires new developments in IoHT environments. One such example^69^ is the application of a cloud-based model for the IoT layers of innovative security solutions using Amazon Web Services and security certificates at each layer of data management. Cloud computing for MFA in the IoT^70^ can be evaluated in terms of attack defence to determine the weakness, strengths, and limitations of the existing methods from a trust-based environment or knowledge-based perspective. Trust-based environments involve the collection of trusted data (e.g. the credentials of a user) to steer heavy computational resources away from device design, which is optimal for IoT devices as they are resource-constrained. The theoretical development of a hybrid cloud^70,71^ using private and public cloud authentication resources allows sensitive data to be encrypted and stored in the private cloud, while public cloud resources are for users to help interact with the system and authenticate themselves through multi-layer security protocols, enhancing authentication security. Knowledge-based authentication uses a more conceptual approach to identify a user based on something they would know about themselves. Another solution of knowledge-based authentication^60^ explores social context through shared knowledge about a user, such as their social status or relationships to move towards trust-based solutions to reduce static approaches. Although knowledge-based authentication is convenient and often timesaving for a workplace that is proactive and demands accessibility remotely, human errors can affect the accuracy and integrity of a social knowledge-based authentication system.^60^ Researchers often find many misconceptions about the effectiveness of a knowledge-based scheme, as it comes down to human involvement being the weakest link in the pursuit of improving the security of authentication systems and providing robust alternatives. The impact of human interactions^60,70,71^ within the system leads to anomalies in testing results that can skew the feasibility of knowledge-based solutions. In contrast, autonomous systems that remove human interaction in authentication systems provide better grounds for the development of an adaptive MFA to mitigate the known vulnerabilities of static methodologies.

### The challenges of IoHT

The challenges of IoHT are inherently the threats of cyber adversaries looking to breach security and acquire critical information, such as in IoT environments, due to the large volume of interconnected^72^ devices and from the lack of understanding and a poor security posture in relation to best-practice cyber security. Knowledge-based factors (e.g. passwords) are common in workplaces, such as hospitals, which have multiple departments and are often interconnected. Thus, patients’ health data might be shared among medical staff and treatment teams, increasing the risk of information leakage and/or oversights with default accounts.^73^ The challenge associated with sharing patient information introduces opportunities of error or risks to cyber security in hospitals and clinics. It is therefore important to ensure MFA is implemented by adhering to privacy protocols and principles. Each medical staff member should be aware of their responsibilities and the consequences of not observing the rules. Healthcare industries may have access to a variety of security options to incorporate MFA into daily operations, with access control tailored to the increase in the attacks from cyber adversaries. However, there are many cultural barriers that hinder the deployment and feasibility of applying MFA solutions in the healthcare sector. It is worth emphasising that adding extra layers of security could increase the complexity of authentication systems, which in turn leads to bad practices by users.^3^ For example, by integrating easy-to-use and easy-to-understand technology such as RFID cards/scanners, users can improve their security posture in the workplace.^3^ Kang et al.^38^ also discussed the value of ‘per-tag’ application, which is beneficial for utilisation in medical environments considering the risk of delay in treating patients. The per-tag technology allows for a single session to be registered to a security card when being used for authentication to reduce the chances of duplication of a legitimate user.^38^ Given that the nature of medical treatment is to supply fast, extensive care to patients, any delay from cyber technologies can lead to excessive costs in damages both financially and to the reputation of the healthcare organisation.

#### Cost of cyber threats to IoHT

This section discusses real-world examples to demonstrate the importance of cyber security and how MFA plays a key role in preventing cyber criminals from gaining access to entry points and breaching the privacy of patients’ sensitive and critical information. Research and development in the IoHT are in high demand, not only for robust and lightweight solutions to authentication systems but for security purposes to address the rising costs of healthcare-related cybercrimes. Cybercrime Magazine^74^ estimates that the cost of cybercrime worldwide could increase to $10.5 trillion by 2025. As a result of the coronavirus pandemic, there has been a surge of people working from home across the world. In the USA, nearly half their workers work from home according to Cybercrime Magazine,^74^ meaning more data is available over cloud networks, and targeted by adversaries. The evidence of the literature reviewed in this paper shows that data breaches commonly occur due to compromised user credentials (e.g. patient personal and medical data), with IBM^75^ reporting that at the entry-level, data breaches accounted for 20% of their findings. According to the Australian Cyber Security Centre (ACSC) report^76^ in 2021, over 1500 reported malicious cyber-attacks were related to the COVID-19 pandemic, disrupting the healthcare sector, which is the second-most targeted industry for ransomware and overall security incidents. Both the 2020 and 2021 reports^76,77^ state that supply chains for the vaccine and medical equipment/supplements were hit by attackers. More importantly, there were serious impacts on critical infrastructures such as hospitals’ local networks, resulting in medical staff being unable to access patient records leading to a service disruption or delay to treatment. Breaches to healthcare systems not only result in financial damage but they also have ethical implications. Cyber criminals can threaten the livelihood or even the survival of patients who need access to health services when time is a critical factor. Information in the 2020 ACSC report can shed light on the 2021 findings^76^ in that attackers’ target people who work from home or use remote access, since often, these users use poorly secured systems, or they do not use MFA. In Germany, it was reported that a patient died due to ransomware attacks on the hospital computer network, which caused ambulances to be re-routed.

Data breaches are common in the healthcare sector because medical records and patient data are often sought by cyber criminals. Data breaches resulted in an alarming cost of $3.86m for healthcare organisations.^3^ According to an IBM report,^75^ data breaches cost the healthcare sector $9.23m, the highest cost of cyber-attacks due to the remote working-from-home response to the pandemic. IBM^75^ also reported that the cost of data breaches hit record highs during the pandemic in 2021, being as much as $4.96m per breach, which increased by an average of $1m due to the remote working factor. An examination of the cause of these breaches indicates that compromised credentials are the root cause of data leaks, where username/passwords are hacked to divulge sensitive information from records such as names, emails, and passwords.^75^ According to the Federal Information Security Modernization Act of 2014, at least 65% of cyber threats to the healthcare industry would have been preventable if better MFA security^3^ had been in place. The statistics^78^ show that hospitals accounted for 30% of large data breaches, and in total, the cost to healthcare organisations in terms of security breaches reached $7 trillion by the end of 2020. While ransomware is not a new concept or threat to the healthcare industry, many organisations have found themselves falling victim to the increased number of attacks. The attacks are reported to be the cost of which increased from an average of $10,000^79^ in 2017 to an average of $100,000 in 2019. These statistics show that there is a greater need for research communities to focus their attention on improving the security approaches of healthcare organisations.

#### Why we need MFA in IoHT

MFA is the frontline defence that attackers must overcome to start any damage. Healthcare-related attacks can be launched against medical professionals and patients as well as the medical data being handled, stored, or transmitted. As user authentication is the entry point for cyber criminals or malicious actors,^62^ the security efforts of the healthcare sector can be at risk^80^ if access control is not properly configured, maintained, or deployed.

Confidentiality, integrity, and availability are the core principles of security in the application of MFA. With MFA, hard-to-replicate factors, such as biometric recognition, can be implemented in the IoHT. Managing IoHT security for authentication and identity handling requires that patient data be kept safe and access control be conducted appropriately with best-practice guidelines and user awareness to prevent the risk of a data breach. The healthcare sector is often resource-constrained, so cyber security funding can be overlooked^80^ when planning secure authentication strategies. The issue in the IoHT, especially the authentication issue, is that the considerable number of interconnected IoT devices in hospital departments and medical facilities expands the attack surface that needs to be covered by rules and policies to prevent and remediate cyber incidents.^80^ Patient healthcare information handled by medical staff needs to be accessed securely and legally protected from unauthorised users. That is why rules or rights^81^ to a user's account should be set for authentication purposes. To have strong IoHT security, ease-of-access and trust of the product are a necessity in the development; seeking to provide a solution to ensuring privacy in authentication, without complexity in design.^82^ The privacy of sensitive or confidential data must meet legal, social, and ethical guidelines when MFA solutions to the IoHT^82^ (e.g. wearables) are developed. MFA systems in IoHT settings^66^ that allow patients to utilise health services remotely, outside of the facilities of a medical practice, clinic, or hospital, need to conform to guidelines and ethical procedures.

The benefits of MFA not only relate to security but also relate to workplace efficiency,^3^ allowing medical staff to access patients’ records, dispensary systems for medicines or live data from sensors/monitors in real time. Software-centric and cloud-based authentication systems^83^ can handle resources over logical distributed networks to check for MFA components without additional physical or hardware requirements. With a distributed system, it is possible to replace simple login scenarios, where an attacker can impersonate a legitimate user using stolen credentials. Cloud-based MFA can help to reduce the management of access control and have security protocols implemented^84^ to fend off attacks. Securing healthcare data is a priority for future researchers, given that there is a fast-growing market for the development of robust MFA to meet the requirements of the IoHT.^34^ Existing MFA systems in IoHT environments are facing challenges from cyber threats^73,80^ and lack prevention and mitigation strategies (e.g. attacks on communication channels).

### Existing solutions for IoHT MFA

In this section, the existing solutions for current MFA systems in IoHT are categorised based on accepted factors and potential authentication systems for future research and development. The following solutions are selected as methodologies that were identified by their relevance to this paper. Therefore, many solutions exist in MFA applications for IoT environments, but for the purpose of identifying key security requirements in healthcare, the following are provided as recommendations. We suggest the following solutions based on their comprehension and advancement towards better security options against traditional passwords or SFA components.

#### Web-authentication solutions

Most devices in the IoHT allow users to interact with health services or systems through a web service or portal system on the intranet, which cannot be accessed by medical staff remotely without the use of the Internet. Fast Identity Online and WebAuthn^85,86^ perform user authentication by removing the need for a password using public/private key cryptography, making it a time-saving solution. Private keys are stored in a secure environment, while the user has the public key tied to an authenticator on a device, such as a physical key device.^86^ The FIDO2 protocol further develops this password-free approach with industry-known physical key devices generating private keys. The security measures ensure that even the user cannot export the private key. Digital signatures can then be applied as an additional factor with the click of a button on the device when used with WebAuthn services.^85^

#### Biometric solutions

The refinement of smartcards for remote user authentication can be used in combination with biometric authentication systems to improve overall security requirements. Tritilanunt^87^ proposed a biometric solution that was more resilient against common password authentication attacks in physical smart cards.^87^ System security should be thoroughly investigated, as there are many vectors by which attackers can attempt to compromise security keys. As a form of authentication and securing users’ confidential information (e.g. fingerprints), biometric scanners are found in most IoT devices,^45^ such as mobile phones. Biometric authentication involves one or several biometrics traits, such as those used in MFA. Biometric recognition is an appealing alternative to traditional authentication methods (e.g. passwords), which have a higher risk of being compromised. It is much easier for a remote attacker to masquerade their way through a password-based authentication system when they have stolen a user's password. Also, there is no active monitoring of who is behind the device accessing the information.^45,88^ With biometric systems, sensory devices such as fingerprint scanners can be combined with human monitoring to ensure that the identity of a user is verified more reliably.^44,89^ Moreover, biometric authentication can enable users to identify themselves in open environment settings, such as a public hospital. It is becoming more important to have lightweight MFA^61^ for healthcare, as it reduces the time to scan biometric traits and requires no additional hardware.

#### Physical key solutions

Hardware authentication devices, also known as physical key authentication, are a possession factor for users to prove the legitimacy of their identity. In recent years, Universal 2nd Factor security keys^90^ have gained popularity as a lightweight, easy-to-access security option in MFA, which can mitigate the risk of phishing and MITM attacks. 2FA provides a secure, easy-to-use approach for medical staff^81^ who need to access patients’ records frequently. RFID is a popular authentication methodology to meet the security and cost-effective requirements for the expansion of IoHT devices^36,38^ in medical contexts. More specifically, as a physical key solution to providing an additional layer of security for medical practices,^91^ it is necessary for RFID to maintain its lightweight paradigm in future research and development. Because RFID and NFC^36^ are based on close-proximity usage for identification, many IoHT devices are equipped with such technology, which is desirable in the healthcare environment as contactless options have been particularly promoted during the COVID-19 pandemic.

**Figure 6.**
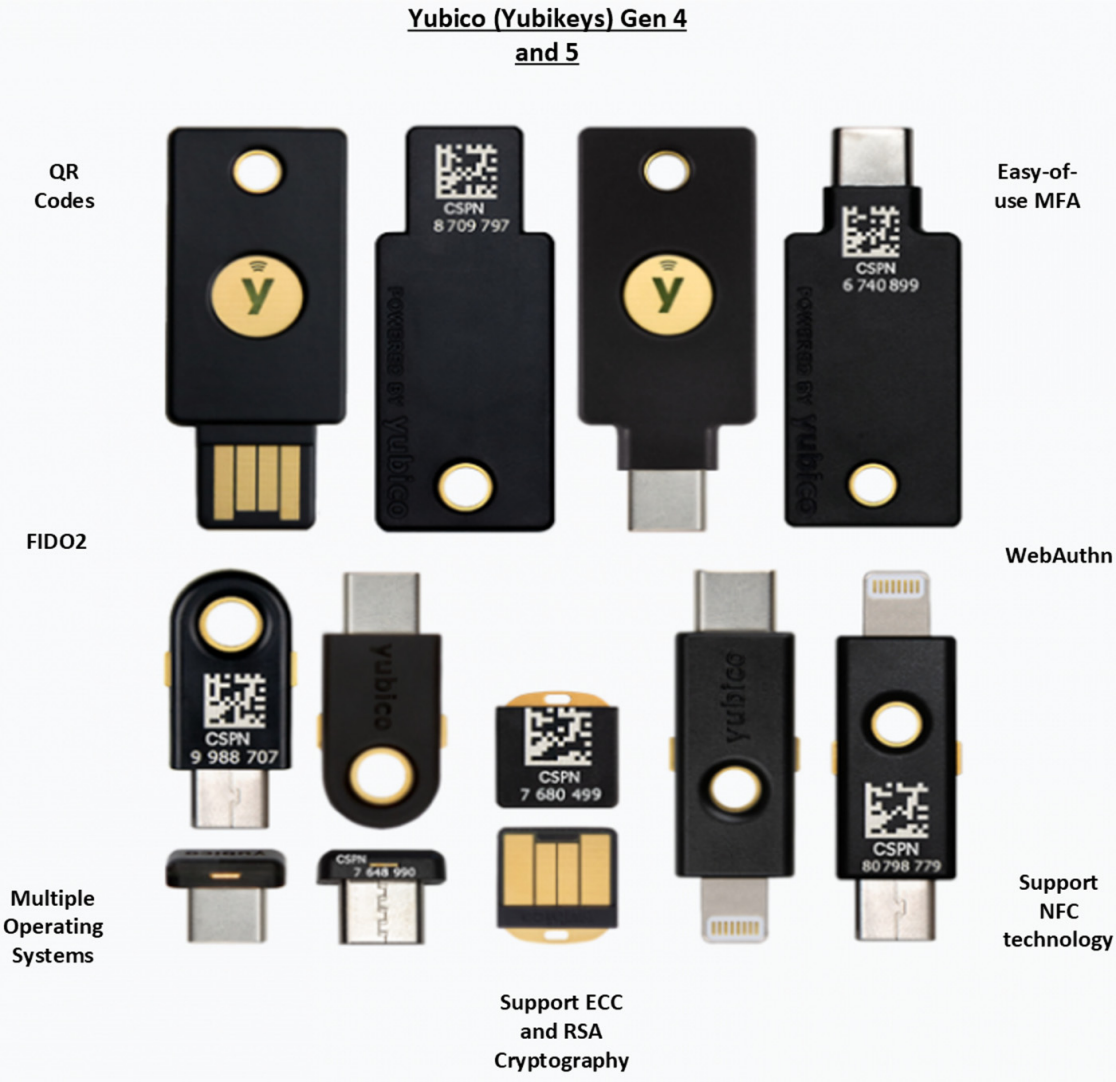
Popularphysical security keys available for current authentication solutions for possession-based MFA.^92^

YubiKeys^92^ have been widely discussed in their application to MFA and are suited as secure solutions to various cyber threats. YubiKeys provide the security of an OTP in the form of a physical device, which can wirelessly communicate with systems requiring authentication. YubiKeys are favourable in the healthcare setting in that medical staff can reduce authentication time when performing tasks that are repeated continuously throughout the day. Studies^90^ have found that users prefer physical keys to mobile devices or checking for OTPs. Yubico, the distributor of these physical keys, is releasing advanced iterations of YubiKeys, with recent developments including biometric scanners.^90,92^ There are many available solutions for physical security keys on the market, for both commercial and personal use suitable for healthcare workers and patients accessing e-health data from home, as shown in Figure 6.

#### Cloud-based solutions

Cloud-based solutions allow patients’ data and medical information to be accessed without the restriction of being in a physical location. This means that cloud-based authentication systems can be used while on the move, a desired property for healthcare services.^93^ Cloud computing is a practical option for MFA. Often organisations outsource computational requirements to cloud computing platforms, which handle enormous amounts of data. Therefore, it is important that MFA is incorporated with best practices to ensure the confidentiality, availability, and integrity of sensitive data, while rendering robust and lightweight authentication solutions.^94^ With the vast amounts of data and resources in cloud services, adversaries can compromise the integrity of authentication systems if security is an oversight.^71^

Cloud-as-a-Service authentication systems^83^ can be deployed to offset the high running costs of organisations with hardware and maintenance requirements. A hybrid cloud service allows patients to access health services through public cloud systems, which is cost-effective. Cloud-based systems^83^ offer authentication via software control (e.g. digital signatures), and can cater for universal 2FA options, such as physical keys or web authentication methods. Telehealth has emerged in recent years, as patients were not allowed to physically attend health services due to COVID-related restrictions. Cloud-based authentication is beneficial for distributing resources.^95^ Cloud-based systems are developed to combat known attacks through mutual authentication, allowing users to upload and receive medical information from home, while reducing the cost and hardware requirements of traditional authentication systems commonly seen in healthcare environments. These changes help to adapt security needs and ensure that resilience in compliance strategies is a priority in the future development of cloud-based solutions.^71,93,95^

## Research limitations

In this review paper, it is acknowledged that there are limitations of the research and imperfection in the summarisation of MFA in the IoHT. To manage the validity of this review, we make sure that articles published in the past decade were selected from a wide range of reliable sources. As per the ‘Research criteria’ section, we adopt search engine parameters for the scope of papers related to MFA in the IoHT. The summarisation and categorisation of MFA are subject to healthcare practices, and the objective is to review the security requirements of next-generation authentication. We find that it is a non-trivial task to incorporate MFA into the IoHT due to a lack of standard frameworks addressing this task. Therefore, we have approached each individual field of the IoHT domain and reviewed those important and relevant papers. Despite the authors’ expertise in authentication-related research, due to the broad spectrum of MFA, there may be some aspects not fully elaborated on.

## Conclusion

In this paper, we evaluate the current MFA practices in a medical context, where healthcare services became a prime target for cyber criminals during the COVID-19 pandemic. Cyber-attacks on MFA in healthcare environments are reviewed. This paper identifies and elaborates on the challenges in IoHT by extending awareness of the factors and principles of MFA. We also discuss the limitations and challenges of authentication security. As healthcare moves to online or telehealth services, FIDO2 and WebAuthn technologies and physical key devices combined with biometrics are shown to be better alternative MFA solutions compared to static password usage. Several future research directions are highlighted below.

- *Robust and lightweight authentication security systems are needed in IoHT*: Based on the components discussed in this paper, there is an urgent requirement for novel authentication security systems (e.g. robust and lightweight MFA systems) to replace the use of traditional approaches to MFA in large IoHT networks as there are many users involved, and often many devices need to be configured into the network, creating a larger attack surface for healthcare industries.
- *Password-free authentication regime should be a priority*: To ensure the privacy and confidentiality of sensitive data (e.g. medical records), while supporting the use of IoHT devices, which have the advantage of mobility and low hardware intensive requirements, adapting to a password-free authentication regime should be a priority in the design of future MFA schemes.
- *Exploit desired properties and capabilities of multiple techniques*: It is promising to exploit the desired properties of biometrics as well as the capabilities of physical keys, such as the YubiKey Bio series. Further studies need to address the acceptability and usability of biometric YubiKeys in the healthcare sector. It is useful to determine if privacy and security requirements can be met by the addition of a stronger authentication standard without the challenges of typical 2FA OTP configurations.
